# Endovascular interventions in the perioperative management of the Fontan procedure in a developing congenital heart surgery program

**DOI:** 10.1186/1749-8090-8-S1-O245

**Published:** 2013-09-11

**Authors:** I Polivenok, M Cardarelli, O Buchneva, M Gelatt, J Breinholt

**Affiliations:** 1 Cardiac Surgery and Emergent Cardiology, Institute of General and Urgent Surgery, Kharkov, Ukraine; 2 Congenital Cardiac Surgery, International Children's Heart Foundation, Memphis, TN, USA; 3 Pediatric Cardiology, International Children's Heart Foundation, Memphis, TN, USA

## Background

Efforts to develop congenital heart surgery programs are numerous but few view the concomitant development of interventional cardiology as a critical component of these efforts.

## Methods

A case series describing perioperative complications of the first Fontan procedures performed in a developing congenital heart surgery program.

## Results

Two patients underwent the first Fontan palliations while participating in a development program for congenital heart surgery. Both presented at 32-33 months of age with baseline oxygen saturations of 82-84%. Patient 1 underwent an extracardiac Fontan with a GoreTex conduit larger than the IVC due to limited supplies. Postoperatively, the patient decompensated to a low cardiac output state with elevated Fontan pressures and significant edema that did not resolve despite fenestration creation. Catheterization demonstrated inferior conduit anastomotic stenosis and right pulmonary artery (RPA) obstruction by compression. Angioplasty of the extracardiac conduit distal anastomosis and RPA stent implant reduced Fontan pressures. The patient was extubated in 3 days and discharged home at 21 days. Patient 2 underwent a lateral tunnel Fontan and extubated 2 hours after surgery, but developed desaturation and low cardiac output within 24 hours. Catheterization revealed elevated Fontan pressures and a large SVC to pulmonary vein collateral. After coil embolization, the patient slightly improved, but underwent subsequent catheterization several days later where an Amplatzer duct occluder was used to obstruct significant antegrade flow through a previously banded pulmonary valve resulting in acute clinical improvement.

## Conclusion

Availability of interventional cardiology allowed early intervention to perioperative complications, reducing the need for repeat surgery and facilitating good clinical outcomes. Development of combined congenital heart surgery and interventional cardiology programs can be of great benefit for patients.

